# Association between antibody-mediated immune responses of herpesvirus and osteoporosis: Mendelian randomization study

**DOI:** 10.1097/MD.0000000000045337

**Published:** 2025-10-24

**Authors:** Botong Li, Shengjun Chai, Hong Wu, Xiaoxia Fan, Rong Zhang, Ming Hou, Chunmei Cai

**Affiliations:** a Research Center for High Altitude Medicine, Qinghai University Medical College, Xining, P.R. China; b Key Laboratory of the Ministry of High Altitude Medicine, Qinghai University Medical College, Xining, P.R. China; c Key Laboratory of Applied Fundamentals of High Altitude Medicine (Qinghai-Utah Joint Key Laboratory of Plateau Medicine), Qinghai University Medical College, Xining, P.R. China; d Laboratory for High Altitude Medicine of Qinghai Province, Qinghai University Medical College, Xining, P.R. China; e Department of Veterinary Medicine, Qinghai University, Xining, P.R. China.

**Keywords:** antibody, herpesvirus, immune response, Mendelian randomization, osteoporosis

## Abstract

Recently, several cohort studies and case-control studies have revealed an association between herpesvirus (HV) and osteoporosis (OP), while the underlying mechanisms remain unclear. The HV-triggered immune responses may be significant in this relationship. Elucidating the contribution and mechanisms of HV-triggered immune responses in OP provides novel insights into the pathogenesis of this condition and promising therapeutic or preventive strategies for OP. We performed a 2-sample Mendelian randomization (MR) analysis using publicly available genome-wide association studies statistics. The inverse-variance weighted (IVW) method, MR-Egger regression, simple mode, weighted median, and weighted mode were applied in this MR analysis. Subsequently, we conducted leave-one-out sensitivity analysis, MR-Egger regression, and Cochran’s *Q* test to assess the stability, horizontal pleiotropy and heterogeneity within the MR framework, respectively. The IVW MR analysis showed significant effects of HV phenotypes on OP (anti-herpes simplex virus 1 IgG seropositivity: IVW odds ratio (OR) = 1.06, *P* = .04287; varicella zoster virus glycoproteins E and I antibody levels: IVW OR = 1.10, *P* = .00210; Epstein–Barr virus EA-D antibody levels: IVW OR = 0.86, *P* = .00016; Epstein–Barr virus EBNA-1 antibody levels: IVW OR = 0.92, *P* = .00548; human herpesvirus 6 IE1A antibody levels: IVW OR = 0.90, *P* = .02745). Importantly, the results of other MR analysis except MR-Egger regression were consistent with IVW results. Our analysis indicated that 3 distinct HV antibodies were independently associated with OP in a causal manner, providing new insights into the involvement of viral infections in OP progression via affecting bone density and strength.

## 1. Introduction

Osteoporosis (OP) is a multifactorial disease characterized by bone loss and trabecular microstructure damage, leading to an increased risk of fractures and bone fragility.^[1,2]^ Worldwide, there are more than 8.9 million osteoporotic fractures every year.^[3]^ More than 33% of women and 20% of men older than 50 years suffer osteoporotic fractures at least once in their lifetime.^[4]^ With the increase in population aging, OP has emerged as a public chronic disease with its associated fractures, high disability and mortality, and substantial socioeconomic burden.^[5,6]^ Therefore, elucidating the pathogenesis of OP has become an urgent issue.

OP exhibits a significant correlation with older age, female gender, and a spectrum of nutritional and genetic factors. Additionally, other potential risk factors, encompassing smoking, alcohol abuse, caffeine intake, glucocorticoid therapy, low body mass index, physical inactivity, gastrointestinal disorders, and calcium and vitamin D deficiency, may contribute to the OP pathogenesis.^[7]^ Despite these associations, the etiology of OP is not yet fully understood. In recent years, several cohort and case-control studies have identified herpesvirus (HV) as a risk factor for OP, while the specific mechanism remains to be fully elucidated.^[8–10]^ HVs are pervasive in human populations and are capable of causing a variety of diseases through either latent or lytic infections.^[11]^ The HV family encompasses 9 distinct members: 3 alpha-HVs, namely herpes simplex virus (HSV) type 1 (HSV-1), HSV type 2 (HSV-2) and herpes zoster virus (VZV); 4 beta-HVs including human herpesvirus-6A (HHV-6A), HHV-6B, HHV-7, and human cytomegalovirus; and 2 gamma-HVs, encompassing Kaposi’s sarcoma-associated herpesviruses and Epstein–Barr virus (EBV).^[12]^ Interestingly, the correlation between HV infections and OP may be attributed to genetic determinants of infections and subsequent antibody-mediated immune responses, especially considering recent findings that underscore the role of HV-induced antibody-mediated immune responses in autoinflammatory diseases.^[13–15]^ Further investigation is warranted to elucidate the exact role and mechanisms of HV-induced immune responses in the OP development.

In this context, we conducted Mendelian randomization (MR) analysis, a novel approach that employs genetic variants as instrumental variables (IVs),^[16]^ to explore the causal association between antibody-mediated immune responses to HV infections and OP. We performed a 2-sample MR analysis using data from the genome-wide association studies (GWAS) via applying the inverse-variance weighted (IVW) method, MR-Egger regression, simple mode, weighted median, and weighted mode, to identify distinct HV antibodies that may contribute to OP pathogenesis. Our analysis showed that Anti-HSV-1 IgG, VZV glycoproteins E and I antibody, and EBV EBNA-1 antibody were independently and causally associated with OP, providing novel insights into the OP pathogenesis and promising therapeutic or preventive strategies for the disease.

## 2. Materials and methods

### 2.1. Study design

**Figure 1.**
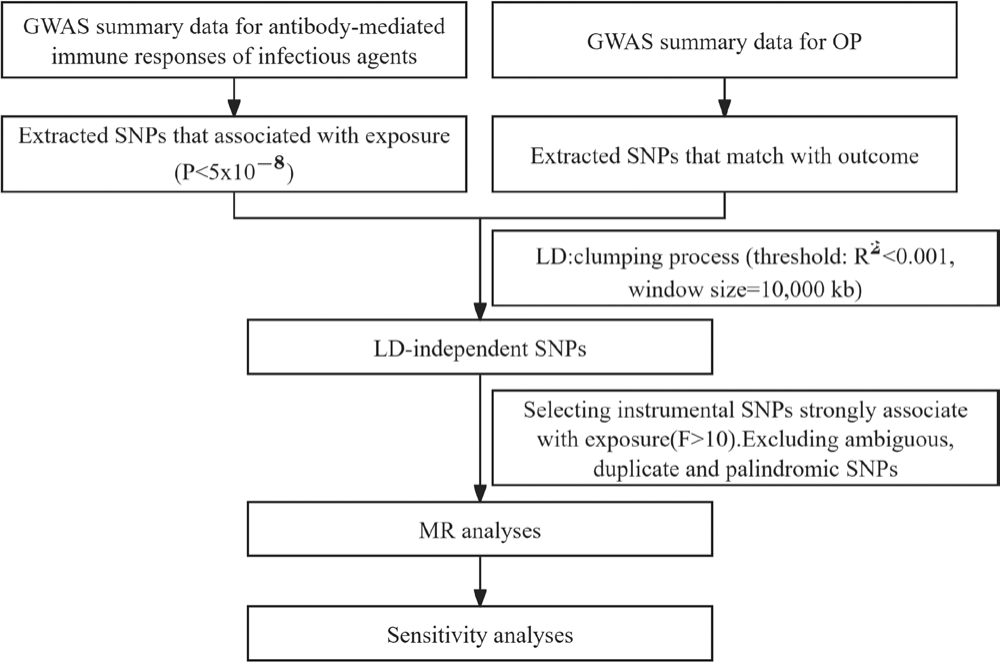
Study design and workflow. GWAS = genome-wide association studies, LD = linkage disequilibrium, MR = Mendelian randomization, OP = osteoporosis, SNP = single nucleotide polymorphism.

We evaluated the causal relationship between 46 phenotypes of infectious agents and OP utilizing a 2-sample MR analysis. MR uses genetic variation to represent risk factors, and therefore, IVs in causal inference must satisfy 3 key assumptions – Relevance assumption: direct association with the exposure. Independence assumption: independence from confounders of the exposure-outcome association. Exclusion restriction assumption: influence on the outcome solely through the exposure.^[17]^ To reduce bias and ensure precise estimation of the associations between modifiable exposures and outcomes, we employed a variety of MR techniques in the research design. The experimental workflow is illustrated in Figure 1.

### 2.2. GWAS data sources for antibody-mediated immune responses of infectious agents

For our analysis of antibody-mediated immune responses to infectious agents, we utilized data from the most extensive available GWAS, by Guillaume Butler-Laporte et al^[18]^ GWAS summary statistics for antibody-mediated immune responses were obtained from the UK Biobank Version 3 imputed genotype data set. There were total of 8735 White British individual participants (55.9% female), identified from a larger pool of 8984 individual samples. There was an average (range) of 4286 (276–8555) samples used for quantitative analyses. The median age (interquartile range) was 58 (51–64) years at the time of enrollment into the UK Biobank. A total of 46 GWAS were performed: 15 case-control analyses and 31 logarithm-transformed MFI analyses. They excluded single nucleotide polymorphisms (SNPs) with minor allele frequencies < 1%.

### 2.3. OP GWAS data sources

GWAS summary statistics for OP were publicly available from the MRCIEU GWAS Catalog (accession numbers from finn-b-M13_OSTEOPOROSIS). This dataset contained 2,12,778 Europeans (3203 cases and 2,09,575 controls) with 16,380,452 SNPs.

### 2.4. Selection of IVs

To ensure the accuracy and effectiveness of our analysis, we imposed stringent inclusion criteria.^[19]^ By recent research, the significance level of IVs for each phenotype and OP was set to 1 × 10^−8^. Secondly, the clumping procedure in 2-Sample MR R package (version 0.5.6) was used to prune these SNPs (linkage disequilibrium [LD] *r*^2^ threshold < 0.001 within 10,000 kb distance) to ensure the independence of the selected IVs and minimize violation of the random allele distribution resulted from LD effects. To match with exposure, SNPs must both satisfy the *P*-value requirement and be free of LD. Thirdly, the *F* statistic (*F* > 10) was calculated for each IV to evaluate IV strength and avoid weak instrumental bias. Strong instruments were defined as IVs with an *F*-statistic > 10, and weak instruments were defined as those with an *F* < 10. Fourthly, ambiguous, duplicate, and palindromic SNPs were automatically discarded during each study, to make sure the same allele was responsible for both the effect of an SNP on the exposure and the outcome. These carefully chosen SNPs were used as the IVs for subsequent 2-sample MR analysis.

### 2.5. Statistical analysis

This 2-sample MR analysis was performed using R software (version 4.2.2) with 2-Sample MR (version 0.5.6) and MR-PRESSO packages (version 1.0.0).

To evaluate the causal association between 46 phenotypes of infectious agents and OP, IVW was employed in the primary MR analyses. When directional pleiotropy is absent, the IVW method can deliver a relatively stable and accurate causal evaluation by using a meta-analytic approach to combine Wald estimates for each IV.^[20]^ The IVW method, which uses all valid SNPs, is advantageous for increasing statistical power. MR-Egger, simple mode, weighted median, and weighted mode were also used to estimate causal effects. The MR-Egger method can provide a relatively robust estimate without the influence of the validity of IVs, and an adjusted result by existing horizontal pleiotropy via the regression slope and intercept.^[21]^ The simple mode is an unweighted mode of the empirical density function of causal estimation.^[22]^ The weighted median can obtain a robust result when more than 50% of weights came from invalid IVs and reduce the type I error to evaluate a more accurate causal association if horizontal pleiotropy exists,^[23]^ while the weighted mode method can obtain a robust overall causal estimate when the majority of similar individual estimates were from valid IVs.^[24]^ However, compared to the IVW method, the MR-Egger, simple mode, weighted median, and weighted mode have compromised power, as indicated by wide confidence intervals (CI) and would only serve as complementary methods in this study.

### 2.6. Pleiotropy, heterogeneity, and sensitivity evaluation

Considering that the IVW approach may be impacted by outliers or significant SNPs and that it is unable to sufficiently handle horizontal pleiotropy and confounding among SNPs, it was necessary to check for heterogeneity and pleiotropy. A series of sensitivity analyses were conducted to further account for potential pleiotropy. At the end of the MR analysis, the results were subjected to sensitivity analyses such as heterogeneity and horizontal multiple validity tests. Cochran’s *Q* statistic and MR-PRESSO test were used to test the heterogeneity among selected IVs.^[25,26]^ Significant heterogeneity was indicated if *P* < .05. MR-Egger’s method was used as a weighted linear regression with intercepts to assess the presence of horizontal pleiotropy among the IVs.^[21]^ In addition, a leave-one-out sensitivity test was used to assess whether the causal effect was significantly influenced by a single SNP.^[27]^ All results are presented as odds ratio (OR) and 95% CI, and results were considered statistically significant when *P* < .05.

## 3. Results

### 3.1. Instrumental variables selection

According to the screening criteria, a total of 1623 SNPs is identified for 46 phenotypes of infectious agents. Additional information regarding these SNPs was detailed in Table S1, Supplemental Digital Content, https://links.lww.com/MD/Q400. Given that the *F*-statistics for all these SNPs exceeded the threshold of 10, they met our predefined selection criteria and showed no signs of LD (*r*^2^ < 0.001). As a result, these SNPs were not regarded as weak instrumental factors.

### 3.2. Two-sample MR analysis

#### 3.2.1. The protective factor of OP

**Figure 2.**
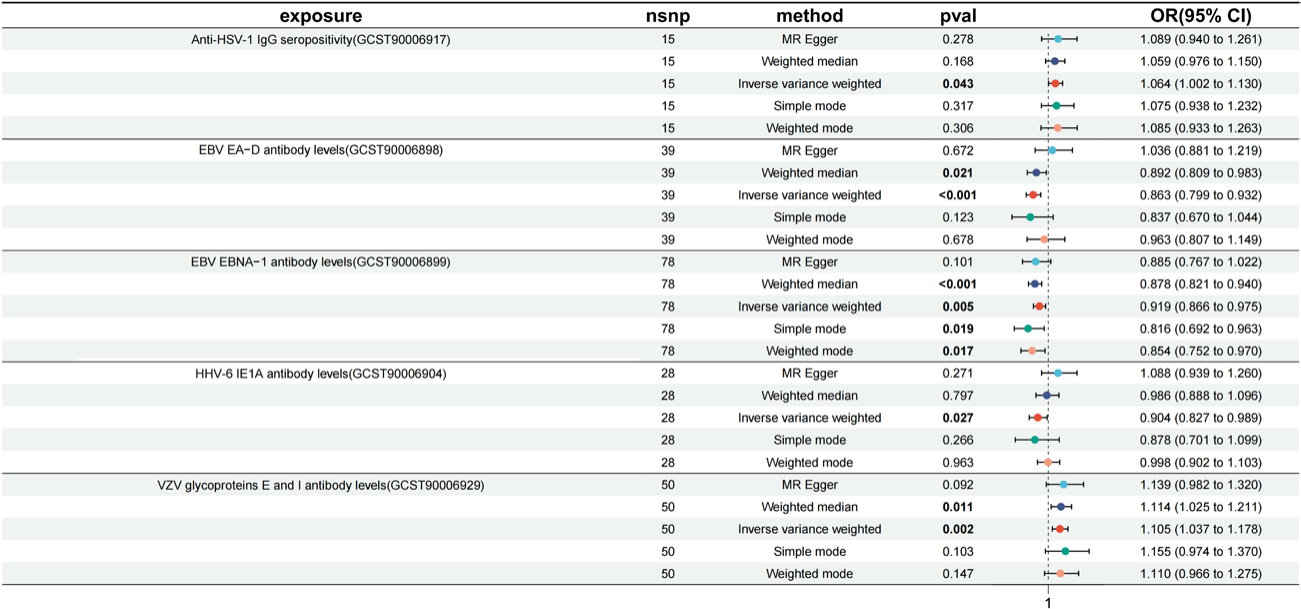
Summary view of the MR estimates between HV phenotypes and OP. Summary of MR analysis results derived from the MR-Egger, weighted median, inverse-variance weighted, simple mode, and weighted mode. EBV = Epstein–Barr Virus, HHV = human herpesvirus, HSV = herpes simplex virus, MR = Mendelian randomization, OR = odds ratio, SNP = single nucleotide polymorphism, VZV = herpes zoster virus.

**Figure 3.**
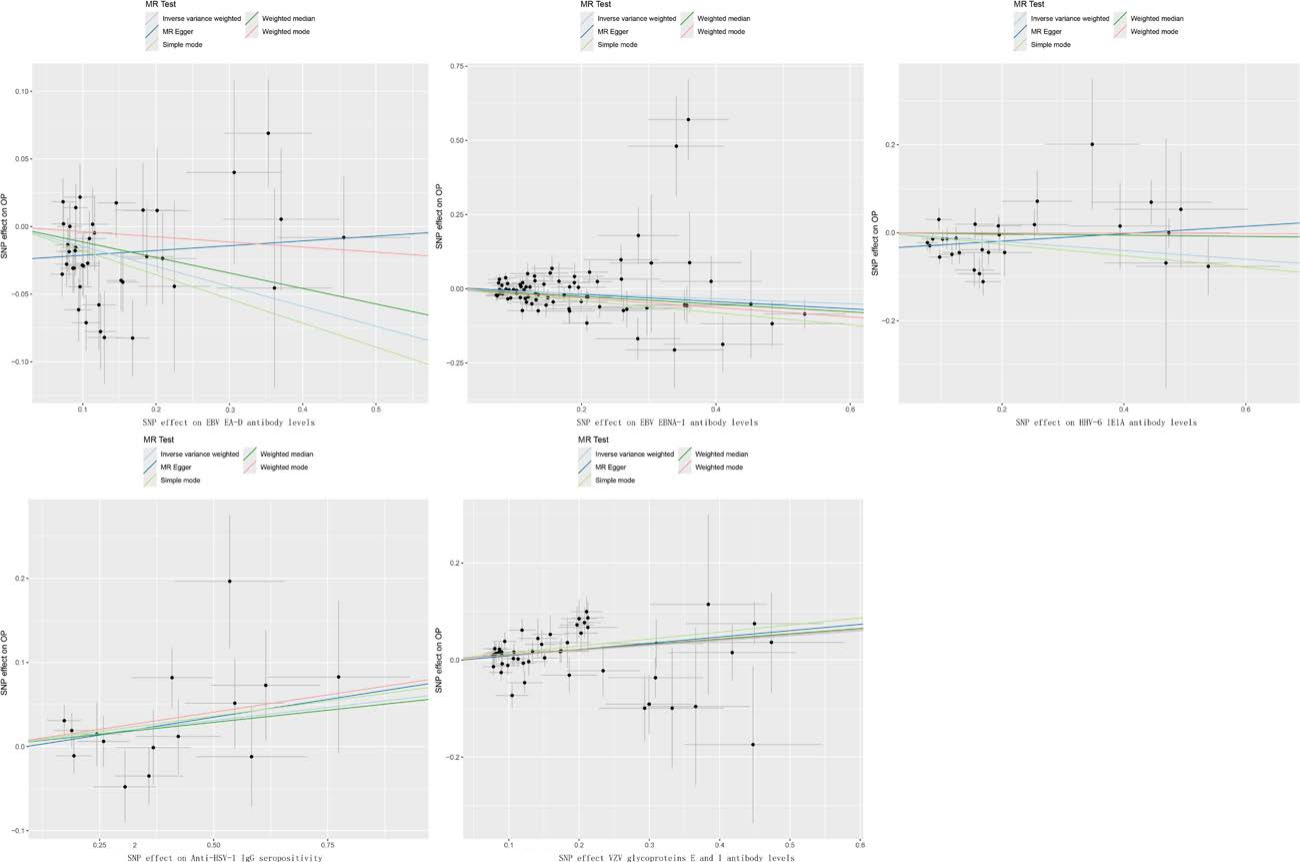
Scatter plots of the causal association between the HV phenotypes and OP. EBV = Epstein–Barr Virus, HHV = human herpesvirus, HSV = herpes simplex virus, MR = Mendelian randomization, SE = standard error.n, SNP = single nucleotide polymorphism, VZV = herpes zoster virus.

As shown in Figures 2 and 3, 5 HV phenotypes were found to be associated with OP in at least one MR method. Among these, 3 phenotypes exhibited a protective effect against OP: levels of EBV EA-D antibody, EBV EBNA-1 antibody, and HHV-6 IE1A antibody. The IVW estimate suggested a protective effect for EBV EA-D antibody levels on OP (OR = 0.86, 95% CI: 0.80–0.93, *P* = .00016), and Weighted median also indicated a protective effect (OR = 0.89, 95% CI: 0.81–0.98, *P* = .02080). The IVW estimate (OR = 0.92, 95% CI: 0.87–0.98, *P* = .00018), weighted median (OR = 0.88, 95% CI: 0.82–0.94, *P* = 2.05 × 10^−3^), simple mode (OR = 0.82, 95% CI: 0.69–0.96, *P* = .01871), and weighted mode (OR = 0.85, 95% CI: 0.75–0.97, *P* = .01745) for EBV EBNA-1 antibody levels consistently s revealed a protective effect against OP, implicating the crucial role of EBV EBNA-1 antibody in impeding OP development. In addition, HHV-6 IE1A antibody levels also showed its suggestive protective effect against OP by IVW (OR = 0.90, 95% CI: 0.83–0.99, *P* = .02745).

Our results were robust even without a potentially influential SNP because of a “leave-one-out” analysis. (Fig. S1, Supplemental Digital Content, https://links.lww.com/MD/Q401) However, heterogeneity assessments were performed using Cochran’s *Q* statistic and MR-PRESSO test, revealing heterogeneity for these 3 protective phenotypes (*P* < .05; Table S2, Supplemental Digital Content, https://links.lww.com/MD/Q400). The funnel plots (Fig. S2, Supplemental Digital Content, https://links.lww.com/MD/Q401) were consistent with these results. In addition, the MR-Egger intercept test demonstrated the horizontal pleiotropy of EBV EA-D antibody levels and HHV-6 IE1A antibody levels (*P* < .05; Fig. 3 and Table S3, Supplemental Digital Content, https://links.lww.com/MD/Q400). Consequently, we excluded the 2 phenotypes (EBV EA-D antibody levels and HHV-6 IE1A antibody levels) and only retained EBV EBNA-1 antibody levels as a protective factor for OP.

#### 3.2.2. The risk factor of OP

Meanwhile, we found 2 phenotypes were detrimental for OP: VZV glycoproteins E and I antibody levels and Anti-HSV-1 IgG seropositivity. Both IVW analysis (OR = 1.10, 95% CI: 1.04–1.18, *P* = .00210) and weighted median (OR = 1.11, 95% CI: 1.03–1.21, *P* = .01107) revealed a significant positive correlation between VZV glycoproteins E and I antibody levels and OP, and the. These findings were consistent with the results of several cohort studies and case-control studies.^[8,9]^ Additionally, Anti-HSV-1 IgG seropositivity was also found to be positively and suggestively associated with OP (OR = 1.06, 95% CI: 1.00–1.13, *P* = .04287).

Similarly, plots of the leave-one-out analysis (Fig. S1, Supplemental Digital Content, https://links.lww.com/MD/Q401) demonstrated that there were no potentially influential SNP driving the causal link and the results were robust. Among these 2 causal associations, the results of Cochran’s IVW *Q* test and MR-PRESSO showed remarkable heterogeneity of VZV glycoproteins E and I antibody levels, whereas no such heterogeneity was observed for Anti-HSV-1 IgG seropositivity (Table S3, Supplemental Digital Content, https://links.lww.com/MD/Q400). The funnel plots (Fig. S2, Supplemental Digital Content, https://links.lww.com/MD/Q401) were also shown to be consistent with the results. Furthermore, neither of the 2 phenotypes showed significant directional horizontal pleiotropy according to the MR-Egger regression intercept analysis (Fig. 3 and Table S3, Supplemental Digital Content, https://links.lww.com/MD/Q400). Therefore, we still regard both phenotypes as risk factors for OP, particularly the Anti-HSV-1 IgG seropositivity.

## 4. Discussion

In this study, we conducted the first comprehensive investigation into the causal relationship between 46 phenotypes of 13 infectious agents and OP using 2-sample MR. We utilized the summary statistics of antibody-mediated immune responses of infectious agents from UK Biobank Version 3 imputed genotype data set and the summary statistics of OP from MRCIEU GWAS. Our analysis revealed evidence for a causal association between 3 HV immunocyte phenotypes and OP, including EBV EBNA-1 antibody levels, VZV glycoproteins E and I antibody levels, and Anti-HSV-1 IgG seropositivity.

EBV EBNA-1 antibody levels exhibited a protective effect on OP, although the exact biological mechanisms for preventing or alleviating OP are poorly understood. Few studies have revealed the potential mechanisms of EBV infection in impeding OP progression. A study has shown that EBV infection leads to upregulation of p62, which in turn activated nuclear factor (erythroid-derived 2)-like 2 via the p62/Kelch-like ECH-associated protein 1/nuclear factor (erythroid-derived 2)-like 2 axis, and the downstream effector glutathione peroxidase 4 was upregulated.^[28]^ This crucial pathway plays a key role in cellular defense against lipid peroxidation and ferroptosis, thus correcting the osteoblast senescence and OP caused.^[29,30]^ In addition, EBV infection might enhance osteogenesis by modulating PPARG RNA decay via YTHDF1, increasing ALPL and osteopontin expression.^[31,32]^

Conversely, our results indicated that VZV glycoproteins E and I antibody levels and Anti-HSV-1 IgG seropositivity were positively correlated with the risk of developing OP. This finding of VZV was in line with several cohort studies and case-control studies.^[8,9]^ The exact underlying mechanisms linking VZV and OP are still not fully understood, while some plausible explanations may be possible. VZV infection could simulate Interleukin-6 (IL-6) production, promoting osteoclastogenesis.^[33,34]^ Furthermore, the elevated levels of IL-6 may enhance autophagy via the IL-6/transcription 3/ATG5 pathway, leading to osteolysis. Collectively, these factors may explain the observed association between VZV infection and an increased risk of OP.^[35]^ Similarly, HSV-1 infection might induce production of cysteine-x-cysteine motif chemokine ligand 1 (CXCL1), CXCL10, and CXCL 11, an inflammatory chemokine, consequently enhancing the osteoclast maturation.^[36,37]^

In brief, this 2-sample MR study identified 1623 SNPs as IVs for the 46 phenotypes, enriching the genetic variants framework. Subsequently, we found the significant positive causal effect of EBV EBNA-1 antibody levels against OP. In contrast, we observed a remarkable negative causal effect of VZV glycoproteins E and I antibody levels and anti-HSV-1 IgG seropositivity. However, this study has limitations: The identification of suitable genetic variants as IVs has become more complex with GWAS data; heterogeneity could introduce bias into MR results; summary statistics rather than raw data limited the exploration of nonlinear relationships and subgroup analyses; the study was based on a European database, and it is debatable whether it applies to other ethnic groups. In conclusion, our study provided a new perspective on the potential role of viral infections in the OP pathogenesis and underscored the importance of considering infectious pathogens as potentially modifiable factors in bone health. Further understanding of how viral infections affect bone density and strength may provide novel therapeutic or preventative strategies for OP.

## Author contributions

**Conceptualization:** Shengjun Chai, Xiaoxia Fan, Ming Hou.

**Investigation:** Chunmei Cai.

**Methodology:** Hong Wu, Chunmei Cai.

**Resources:** Rong Zhang.

**Software:** Chunmei Cai.

**Writing – original draft:** Botong Li, Chunmei Cai.

**Writing – review & editing:** Botong Li.

## Supplementary Material



## References

[1] Xu XM Li N Li K. Discordance in diagnosis of osteoporosis by quantitative computed tomography and dual-energy X-ray absorptiometry in Chinese elderly men. J Orthop Translat. 2019;18:59–64. 31508308 10.1016/j.jot.2018.11.003 PMC6718941

[2] Zhang YW Cao MM Li YJ. The regulative effect and repercussion of probiotics and prebiotics on osteoporosis: involvement of brain-gut-bone axis. Crit Rev Food Sci Nutr. 2023;63:7510–28. 35234534 10.1080/10408398.2022.2047005

[3] The Lancet Diabetes Endocrinology. Osteoporosis: overlooked in men for too long. Lancet Diabetes Endocrinol. 2021;9:1. 10.1016/S2213-8587(20)30408-3 33285120

[4] Shen Y Huang X Wu J. The global burden of osteoporosis, low bone mass, and its related fracture in 204 countries and territories, 1990-2019. Front Endocrinol (Lausanne). 2022;13:882241. 35669691 10.3389/fendo.2022.882241 PMC9165055

[5] Zhu Y Huang Z Wang Y. The efficacy and safety of denosumab in postmenopausal women with osteoporosis previously treated with bisphosphonates: a review. J Orthop Translat. 2020;22:7–13. 32440494 10.1016/j.jot.2019.08.004 PMC7231967

[6] Wang L Yu W Yin X. Prevalence of osteoporosis and fracture in China: the China osteoporosis prevalence study. JAMA Netw Open. 2021;4:e2121106. 34398202 10.1001/jamanetworkopen.2021.21106 PMC8369359

[7] Reid IR Billington EO. Drug therapy for osteoporosis in older adults. Lancet. 2022;399:1080–92. 35279261 10.1016/S0140-6736(21)02646-5

[8] Lin SM Wang CY Chen YY Wang J-H Liang C-C Huang H-K. Herpes zoster and the risks of osteoporosis and fracture: a nationwide cohort study. Eur J Clin Microbiol Infect Dis. 2019;38:365–72. 30460416 10.1007/s10096-018-3436-y

[9] Min C Bang WJ Oh DJ Sim S Choi HG. Association between herpes zoster and osteoporosis: a nested case-control study using a national sample cohort. Biomed Res Int. 2019;2019:4789679. 31467895 10.1155/2019/4789679 PMC6699261

[10] Wu CH Chai CY Tung YC. Herpes zoster as a risk factor for osteoporosis: a 15-year nationwide population-based study. Medicine (Baltimore). 2016;95:e3943. 27336887 10.1097/MD.0000000000003943 PMC4998325

[11] Sathiyamoorthy K Chen J Longnecker R Jardetzky TS. The COMPLEXity in herpesvirus entry. Curr Opin Virol. 2017;24:97–104. 28538165 10.1016/j.coviro.2017.04.006 PMC8601108

[12] Zhong L Zhang W Krummenacher C. Targeting herpesvirus entry complex and fusogen glycoproteins with prophylactic and therapeutic agents. Trends Microbiol. 2023;31:788–804. 36967248 10.1016/j.tim.2023.03.001

[13] Zhang F Zhang B Ding H. The oxysterol receptor EBI2 links innate and adaptive immunity to limit IFN response and systemic lupus erythematosus. Adv Sci. 2023;10:e2207108. 10.1002/advs.202207108 PMC10520634 37469011

[14] Soffritti I D’Accolti M Ravegnini G. Modulation of microRNome by human cytomegalovirus and human herpesvirus 6 infection in human dermal fibroblasts: possible significance in the induction of fibrosis in systemic sclerosis. Cells. 2021;10:1060. 33946985 10.3390/cells10051060 PMC8146000

[15] Fechtner S Berens H Bemis E. Antibody responses to Epstein-Barr virus in the preclinical period of rheumatoid arthritis suggest the presence of increased viral reactivation cycles. Arthritis Rheumatol. 2022;74:597–603. 34605217 10.1002/art.41994 PMC8957485

[16] Greenland S. An introduction to instrumental variables for epidemiologists. Int J Epidemiol. 2000;29:722–9. 10922351 10.1093/ije/29.4.722

[17] Burgess S Davey Smith G Davies NM. Guidelines for performing Mendelian randomization investigations: update for summer 2023. Wellcome Open Res. 2019;4:186. 32760811 10.12688/wellcomeopenres.15555.1 PMC7384151

[18] Butler-Laporte G Kreuzer D Nakanishi T Harroud A Forgetta V Richards JB. Genetic determinants of antibody-mediated immune responses to infectious diseases agents: a genome-wide and HLA association study. Open Forum Infect Dis. 2020;7:ofaa450. 33204752 10.1093/ofid/ofaa450 PMC7641500

[19] Zheng Q Wang D Lin R. Mendelian randomization analysis suggests no associations of human herpes viruses with amyotrophic lateral sclerosis. Front Neurosci. 2023;17:1299122. 38156274 10.3389/fnins.2023.1299122 PMC10754516

[20] Burgess S Butterworth A Thompson SG. Mendelian randomization analysis with multiple genetic variants using summarized data. Genet Epidemiol. 2013;37:658–65. 24114802 10.1002/gepi.21758 PMC4377079

[21] Burgess S Thompson SG. Interpreting findings from Mendelian randomization using the MR-Egger method. Eur J Epidemiol. 2017;32:377–89. 28527048 10.1007/s10654-017-0255-x PMC5506233

[22] Milne RL Kuchenbaecker KB Michailidou K.; ABCTB Investigators. Identification of ten variants associated with risk of estrogen-receptor-negative breast cancer. Nat Genet. 2017;49:1767–78. 29058716 10.1038/ng.3785 PMC5808456

[23] Bowden J Davey Smith G Haycock PC Burgess S. Consistent estimation in Mendelian randomization with some invalid instruments using a weighted median estimator. Genet Epidemiol. 2016;40:304–14. 27061298 10.1002/gepi.21965 PMC4849733

[24] Hartwig FP Davey Smith G Bowden J. Robust inference in summary data Mendelian randomization via the zero modal pleiotropy assumption. Int J Epidemiol. 2017;46:1985–98. 29040600 10.1093/ije/dyx102 PMC5837715

[25] Greco M FD Minelli C Sheehan NA Thompson JR. Detecting pleiotropy in Mendelian randomisation studies with summary data and a continuous outcome. Stat Med. 2015;34:2926–40. 25950993 10.1002/sim.6522

[26] Verbanck M Chen CY Neale B Do R. Detection of widespread horizontal pleiotropy in causal relationships inferred from Mendelian randomization between complex traits and diseases. Nat Genet. 2018;50:693–8. 29686387 10.1038/s41588-018-0099-7 PMC6083837

[27] Cheng H Garrick DJ Fernando RL. Efficient strategies for leave-one-out cross validation for genomic best linear unbiased prediction. J Anim Sci Biotechnol. 2017;8:38. 28469846 10.1186/s40104-017-0164-6 PMC5414316

[28] Yuan L Li S Chen Q. EBV infection-induced GPX4 promotes chemoresistance and tumor progression in nasopharyngeal carcinoma. Cell Death Differ. 2022;29:1513–27. 35105963 10.1038/s41418-022-00939-8 PMC9346003

[29] Dodson M Castro-Portuguez R Zhang DD. NRF2 plays a critical role in mitigating lipid peroxidation and ferroptosis. Redox Biol. 2019;23:101107. 30692038 10.1016/j.redox.2019.101107 PMC6859567

[30] Xu P Lin B Deng X Huang K Zhang Y Wang N. VDR activation attenuates osteoblastic ferroptosis and senescence by stimulating the Nrf2/GPX4 pathway in age-related osteoporosis. Free Radic Biol Med. 2022;193:720–35. 36402439 10.1016/j.freeradbiomed.2022.11.013

[31] Xu YY Li T Shen A. FTO up‐regulation induced by MYC suppresses tumour progression in Epstein–Barr virus-associated gastric cancer. Clin Transl Med. 2023;13:e1505. 38082402 10.1002/ctm2.1505 PMC10713874

[32] Chen L-S Zhang M Chen P. The m6A demethylase FTO promotes the osteogenesis of mesenchymal stem cells by downregulating PPARG. Acta Pharmacol Sin. 2022;43:1311–23. 34462564 10.1038/s41401-021-00756-8 PMC9061799

[33] Jarosinski KW Carpenter JE Buckingham EM. Cellular stress response to varicella-zoster virus infection of human skin includes highly elevated interleukin-6 expression. Open Forum Infect Dis. 2018;5:ofy118. 30014002 10.1093/ofid/ofy118 PMC6007511

[34] Zheng Q Lin R Wang D Zheng C Xu W. Effects of circulating inflammatory proteins on spinal degenerative diseases: evidence from genetic correlations and Mendelian randomization study. JOR Spine. 2024;7:e1346. 38895179 10.1002/jsp2.1346 PMC11183170

[35] Buckingham EM Girsch J Jackson W Cohen JI Grose C. Autophagy quantification and STAT3 expression in a human skin organ culture model for innate immunity to herpes zoster. Front Microbiol. 2018;9:2935. 30568636 10.3389/fmicb.2018.02935 PMC6290052

[36] Korbecki J Gąssowska-Dobrowolska M Wójcik J. The importance of CXCL1 in physiology and noncancerous diseases of bone, bone marrow, muscle and the nervous system. Int J Mol Sci. 2022;23:4205. 35457023 10.3390/ijms23084205 PMC9024980

[37] Zheng Q Wang D Lin R. Effects of circulating inflammatory proteins on osteoporosis and fractures: evidence from genetic correlation and Mendelian randomization study. Front Endocrinol (Lausanne). 2024;15:1386556. 38757000 10.3389/fendo.2024.1386556 PMC11097655

