# Supplementary material for: Association between antibody-mediated immune responses of herpesvirus and osteoporosis: Mendelian randomization study
Source: Medicine (Baltimore). 2025 Oct 24;104(43):e45337. doi: 10.1097/MD.0000000000045337 (PMC12558204; doi:10.1097/MD.0000000000045337)
Supplement: Supplementary file 2 [file medi-104-e45337-s002.docx]

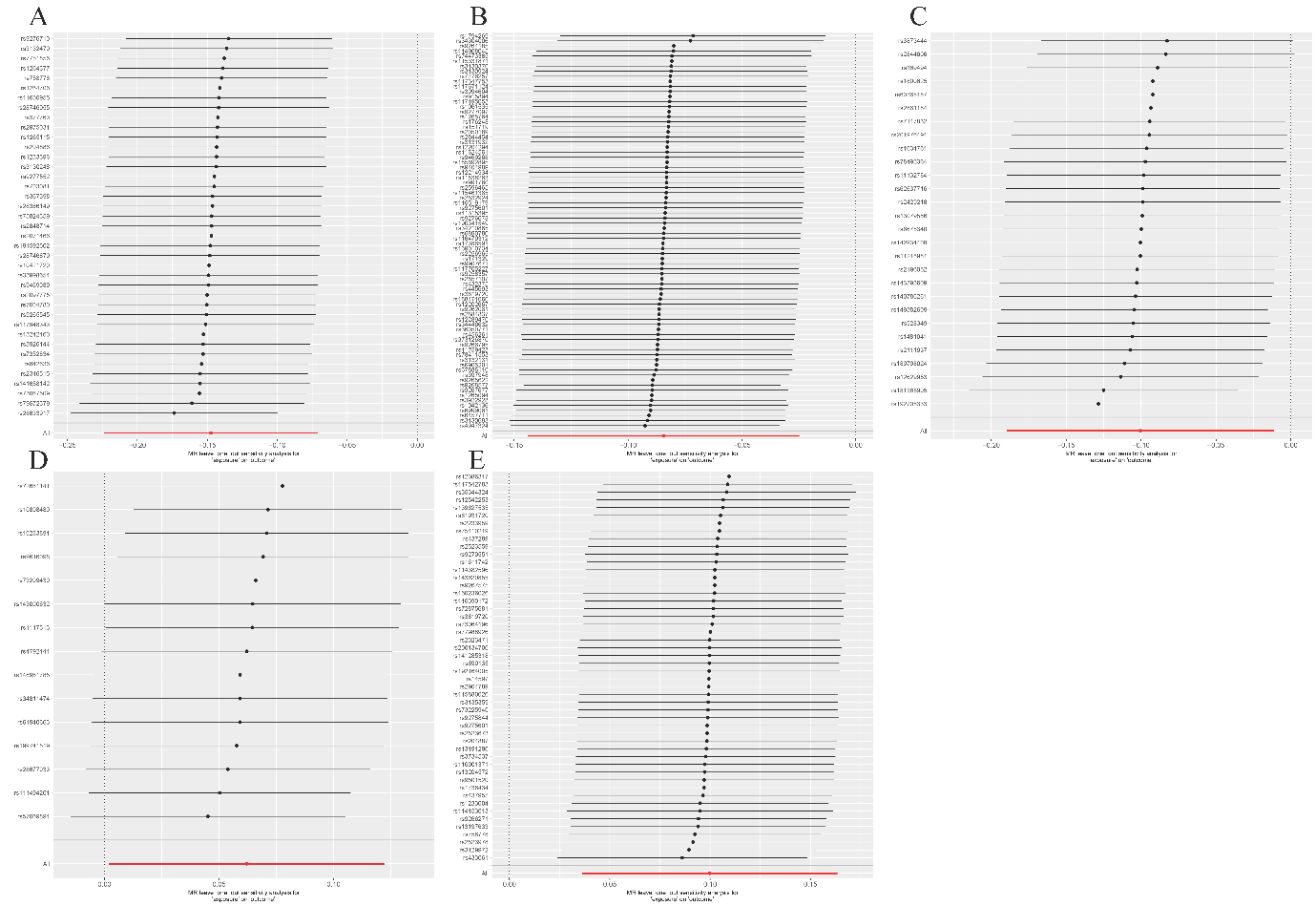


**Figure S1. Leave-one-out analyses for the causal estimates between HV phenotypes and OP. (A) EBV EA-D antibody levels (B) EBV EBNA-1 antibody levels (C) HHV-6 IE1A antibody levels (D) Anti-HSV-1 IgG seropositivity(E) VZV glycoproteins E and I antibody levels.** HV, herpesvirus; OP, osteoporosis ; MR, mendelian randomization; EBV, epstein barr virus; HHV, human herpesvirus; HSV, herpes simplex virus; VZV, herpes zoster virus.


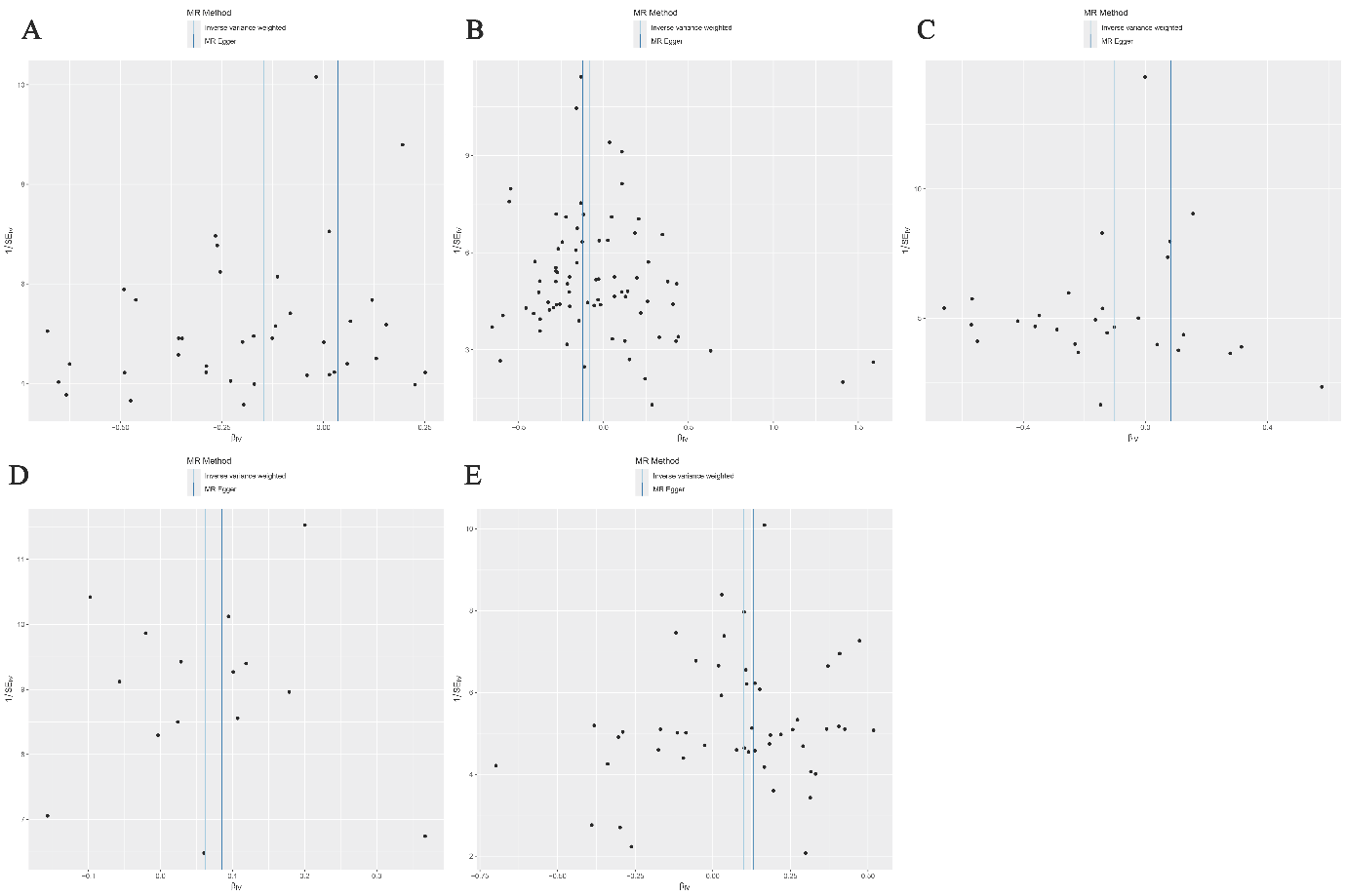


**Figure S2. Funnel plots of the causal association between HV phenotypes and OP. (A) EBV EA-D antibody levels (B) EBV EBNA-1 antibody levels (C) HHV-6 IE1A antibody levels (D) Anti-HSV-1 IgG seropositivity(E) VZV glycoproteins E and I antibody levels.** HV, herpesvirus; OP, osteoporosis ; MR, mendelian randomization; EBV, epstein barr virus; HHV, human herpesvirus; HSV, herpes simplex virus; VZV, herpes zoster virus.
